# Plasma from Volunteers Breathing Helium Reduces Hypoxia-Induced Cell Damage in Human Endothelial Cells—Mechanisms of Remote Protection Against Hypoxia by Helium

**DOI:** 10.1007/s10557-019-06880-2

**Published:** 2019-04-25

**Authors:** Kirsten F. Smit, Gezina T. M. L. Oei, Moritz Konkel, Quinten J. J. Augustijn, Markus W. Hollmann, Benedikt Preckel, Hemal H. Patel, Nina C. Weber

**Affiliations:** 1 Department of Anesthesiology, Laboratory of Experimental Intensive Care and Anesthesiology (L.E.I.C.A), AUMC, Academic Medical Centre (AMC), University of Amsterdam, Meibergdreef 9, 1100 DD Amsterdam, The Netherlands; 2 Department of Anesthesiology, University of California, San Diego and VA San Diego Healthcare System, San Diego, CA USA

**Keywords:** Endothelial conditioning, Remote, Helium, Ischemic preconditioning, Caveolin-1

## Abstract

**Purpose:**

Remote ischemic preconditioning protects peripheral organs against prolonged ischemia/reperfusion injury via circulating protective factors. Preconditioning with helium protected healthy volunteers against postischemic endothelial dysfunction. We investigated whether plasma from helium-treated volunteers can protect human umbilical vein endothelial cells (HUVECs) against hypoxia in vitro through release of circulating of factors.

**Methods:**

Healthy male volunteers inhaled heliox (79% helium, 21% oxygen) or air for 30 min. Plasma was collected at baseline, directly after inhalation, 6 h and 24 h after start of the experiment. HUVECs were incubated with either 5% or 10% of the plasma for 1 or 2 h and subjected to enzymatically induced hypoxia. Cell damage was measured by LDH content. Furthermore, caveolin 1 (Cav-1), hypoxia-inducible factor (HIF1α), extracellular signal-regulated kinase (ERK)1/2, signal transducer and activator of transcription (STAT3) and endothelial nitric oxide synthase (eNOS) were determined.

**Results:**

Prehypoxic exposure to 10% plasma obtained 6 h after helium inhalation decreased hypoxia-induced cell damage in HUVEC. Cav-1 knockdown in HUVEC abolished this effect.

**Conclusions:**

Plasma of healthy volunteers breathing helium protects HUVEC against hypoxic cell damage, possibly involving circulating Cav-1.

**Electronic supplementary material:**

The online version of this article (10.1007/s10557-019-06880-2) contains supplementary material, which is available to authorized users.

## Introduction

Noble gases like helium [1, 2] and xenon [3–5] can induce preconditioning and protect organs against ischemia-reperfusion (I-R) injury. The mechanism behind helium preconditioning remains unclear, but involvement of the reperfusion injury salvage kinase (RISK) pathway and its downstream targets [6, 7], the mitochondrial adenosine triphosphate–regulated potassium channel (K^ATP^) [8] and calcium sensitive potassium channel have been described [9, 10]. These mediators are all located on the mitochondria, suggesting that the mechanism of helium conditioning might congregate on this level.

Caveolins are structural proteins that are essential in the formation of cholesterol- and sphingolipid-enriched invaginations of the plasma membrane called “caveolae” [11, 12]. Caveolae, a subtype of lipid rafts, contain a scaffolding domain that anchors and regulates proteins and influences cellular processes, including vascular transport and signal transduction [12]. These signalling molecules that aggregate into multiprotein complexes known as signalosomes are continuously forming and dissociating under basal and stimulated conditions and are regulated by caveolins [13].

Many of the protein complexes involved in organ protection via preconditioning are known to bind to the scaffolding domain of caveolins and are regulated by caveolins [14, 15]. As such, caveolins are critically involved in ischemic preconditioning [16].

Ischemic preconditioning not only protects against ischemia-reperfusion injury on a local level in multiple organs, but also protects remote tissues, a phenomenon known as remote ischemic preconditioning (RIPC) via release of circulatory protective factors [17]. It was shown that plasma obtained from healthy volunteers undergoing RIPC protects against hypoxia-induced damage in endothelial cells, suggesting involvement of a potential humoral mediator for protection [18]. Whether noble gases mediate a release of humoral factors to initiate an RIPC-like effect is yet unknown.

A previous in vitro study showed that helium treatment of endothelial cells indeed lowers levels of caveolin-1 and increases levels of circulating caveolin-1 in the supernatant of these cells [19]. Such data support the hypothesis that circulating factors in the blood stream may be involved in inducing organ protection by helium.

All blood vessels are lined by a layer of endothelial cells, and the endothelium is the first contact site for a humoral mediator of RIPC. The endothelium may interact with the mediator and either directly or indirectly transfer the RIPC stimulus to the target organ. We hypothesised that plasma collected from human volunteers exposed to helium inhalation contains a helium-induced release of cellular factors containing cell protective features and that caveolins may be central to this effect. As the endothelium is the first contact site for a remote stimulus, we investigated whether plasma from human volunteers would protect against hypoxia in endothelial cells.

## Methods

The study was approved by the ethical committee of the Academic Medical Centre, Amsterdam (http://www.trialregister.nl/NTR4507) and was conducted in accordance with the International Conference on Harmonisation on Good Clinical Practice Guidelines and the Declaration of Helsinki.

After obtaining informed consent, a total of 20 healthy, non-smoking, male volunteers (age 20–55) were included in this explorative crossover study. Exclusion criteria for the volunteers were any form of chronic disease, smoking and history of allergic reaction to medication. Baseline levels of haemoglobin, leucocytes and C-reactive protein were determined before each experimental cycle, and no difference was observed between groups (see Table 1). All volunteers were treated in the Academic Medical Centre Amsterdam from 16 June 2014 until 21 August 2014. One volunteer did not complete the study and was removed from analysis.

**Table 1:** Biochemical data of volunteers

	Control gas	Helium
CRP (mmol/l)	1.1 ± 1.3	1.3 ± 1.9
Haemoglobin (mmol/l)	9.2 ± 0.5	9.2 ± 0.5
Leucocytes (mmol/l)	5.2 ± 0.9	5.5 ± 1.0

Data are represented as means ± SD. No significant differences were observed between cycles

*CRP* C-reactive protein, *Hb* haemoglobin, *L* leucocytes

Thus, all observations reflect the results of *n* = 19 besides the knockdown experiments. However, for the knockdown experiments (Fig. 4), we focused on the primary outcome of the study, namely the protection of plasma against the hypoxia-induced cell damage, and at the time point where the initial protection was seen (Fig. 2). The knockdown experiments were very resource consuming and took most of the plasma we had left, so based on this, we were not able to measure LDH in the knockdown cells at the time points 30 min and 24 h.

**Fig 1:**
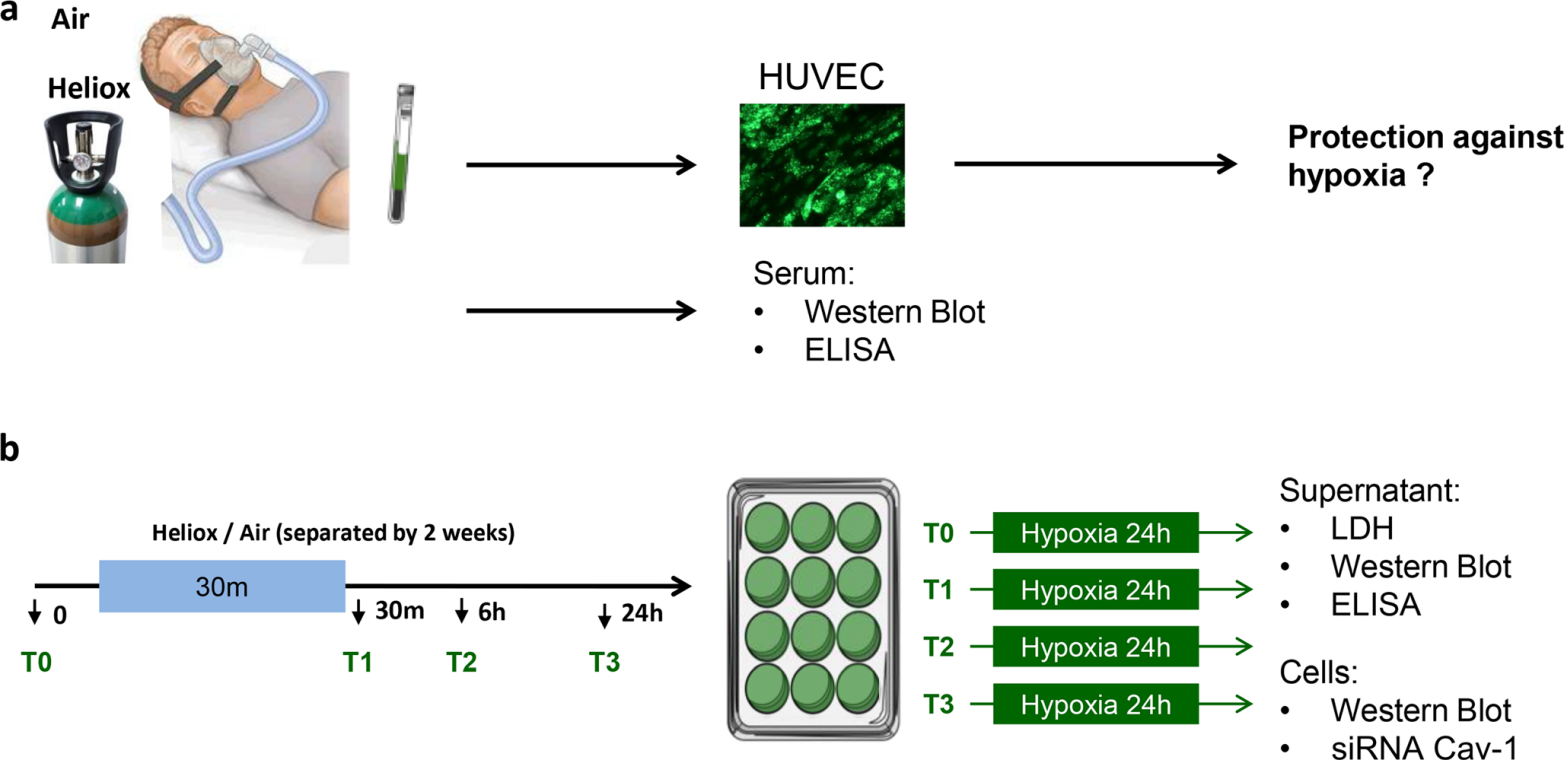
Protocol outline. Healthy volunteers received either helium or control gas via face mask in 2 cycles (separated by 2 weeks). Blood samples were taken at baseline (T1) and 30 min (T2), 6 h (T3) and 24 h (T4) after treatment (**b**). HUVECs were incubated with plasma and subjected to 24 h of hypoxia, after which cells and supernatant were harvested for analysis

Experiments were performed in a quiet, temperature-controlled room. Twelve hours prior to the start of the experiments, volunteers refrained from alcohol or caffeine-containing drinks and were denied heavy physical exercise. All volunteers underwent two experimental cycles (separated by 2 weeks), breathing either 30 min of heliox (79% He/21% O_2_, BOC, Mordon, UK) or air using a non-invasive delivery system via a normal face mask with pressure support of 3-cm H_2_O. None of the volunteers had any sign of dyspnoea or any kind of stress; they were all normally breathing and calm. Blood was collected before (baseline, T0) and directly after inhalation of heliox or air (T1), as well as 6 h (T2) and 24 h (T3) after start of the experiment by separate venous punctures (see Fig. 1). Samples were collected in citrate vials (BD Bioscience, Breda, the Netherlands) and stored at − 80 °C after centrifugation (290×*g* for 10 min at 4 °C).

### Cell Culture

HUVECs were collected from human umbilical veins as described previously [19] (Waiver W12-167#12.17.096, Ethical Committee AMC, Amsterdam). Experiments were performed with cells of passages 3 and 4. HUVECs were identified using antibodies against von Willebrand factor (data not shown).

### Enzymatically Induced Hypoxia

Hypoxia was induced by enzymatic oxygen depletion using 4 U/ml glucose oxidase and 120 U/ml catalase as previously described [18]. For this study, the concentration of hypoxia-inducing enzymes was optimised after measuring oxygen levels in cell culture medium using the OxyMini fibre optic oxygen meter (World Precision Instruments, USA, Sarasota) and OxyMicro Software v.00 04/2003. After hypoxia induction, cell culture plates were put in an airtight chamber (Billups-Rothenberg, Del Mar, CA, USA), flushed with nitrogen until the oxygen concentration was < 1% and kept in the incubator at 37 °C for 24 h.

### Experimental Protocol

Upon cell confluence, HUVECs were seeded onto gelatine-coated 12-well plates 2 days prior to start of the subsequent in vitro experiments. Before the start of enzymatically induced hypoxia, cells were incubated at 37 °C for 1 or 2 h with normoxic M199 (PAN Biotech, Aidenbach, Germany) containing 5% or 10% of the plasma samples of the volunteers (percentage as diluted in cell culture medium, each four time points of the two experimental cycles; see Fig. 1). Subsequently, this medium was replaced with hypoxic M199, and respective plasma and plates were put in an airtight chamber for 24 h. Cell culture medium of each well was collected and frozen at − 20 °C after 24 h of hypoxia for colorimetric lactate dehydrogenase (LDH) activity measurements and enzyme-linked immune assay (ELISA). Cells were harvested and frozen at − 80 °C for western blot analysis. Additional experiments were performed using small interfering RNA (siRNA) Cav-1-transfected HUVEC (*n* = 10).

### Lactatdehydrogenase Measurement and Enzyme-Linked Immune Assay

In the supernatant, LDH activity was measured by colorimetric analysis using LDH kit (Biovision, Uithoorn, the Netherlands) according to the manufacturer’s protocol. An ELISA reader (Tecan, Crailsheim, Germany) was used to measure light absorbance. Plasma levels of caveolin-1 (BlueGene, Shanghai, China) and eNOS (R&D Systems, Minneapolis, USA) were determined by ELISA.

### Western Blotting

Western blotting was performed as described previously [19, 20]. In order to obtain all membrane-bound proteins in the cell lysate, we performed ultrasonic lysis by sonification with each sample. Overnight incubation with primary antibodies was performed for caveolin-1 (Abcam, Cambridge, UK 1:20,000); glyceraldehyde 3-phosphate dehydrogenase (GAPDH) (Abcam, Cambridge, UK, 1:5000); HIF1α (Acris, Novus Biological, UK, 1:1000); phosphoERK1/2 (Cell Signalling, Danvers, USA, 1:5000), ERK1/2 (Cell Signalling, Danvers, USA, 1:5000), phospho-eNOS and eNOS (Cell Signalling, Danvers, USA, 1:1000), phosphoSTAT3 (R&D Systems, Minneapolis, USA, 1:1000) or against STAT3 (R&D Systems, Minneapolis, USA, 1:1000).

Membranes were washed for 3 × 5 min in tris-buffered saline containing Tween buffer (TBST) before incubating with the appropriate secondary antibody (IRdye, LI-COR, Bad Homburg, Germany) for 1 h at room temperature. After a final washing, membranes were scanned with the Odyssey infrared imaging system (LI-COR, Bad Homburg, Germany), and quantification of the signals was performed with Odyssey Imaging Studio software (LI-COR, Bad Homburg, Germany).

### Transfection with siRNA for Cav-1

We transfected HUVEC at a confluency of 50–80% with siRNA for Cav-1 (caveolin-1 (sense) sequence (5′-3′) CCCUAAACACCUCAACGAU(dT)(dT), Cav-1 (antisense) sequence (5′-3′) AUCGUUGAGGUGUUUAGGG(dT)(dT), Sigma-Aldrich, Zwijndrecht, The Netherlands) or negative control siRNA (Silencer® Negative Control siRNA, Ambion by Thermo Fischer Scientifics, Waltham, MA, USA) using Lipofectamine RNAiMax (Invitrogen by Thermo Fischer Scientifics, Waltham, MA, USA). siRNA for Cav-1 or negative control siRNA was used at a final concentration of 100 nM on the cells and was left on the cells for 24 h. After 72 h, cells were lysated to determine protein knock down by western blot analysis or experiments were started.

### Statistical Analysis

All data are shown as mean ± 95% CI. Statistical analyses were performed using GraphPad Prism 7.0 (La Jolla, CA, USA) software. Data were analysed for normal distribution using the D’Agostino test. Student’s *t* test was used to analyse parametric data (WB data, ELISA), and two-way ANOVA was used to analyse LDH activity. To avoid risk of bias, all investigators performing the laboratory analyses worked with coded thus blinded samples. *p* < 0.05 was considered significant.

## Results

### Prolonged Exposure to Helium Plasma Reduces Hypoxia-Induced Cell Damage in HUVEC

**Fig. 2:**
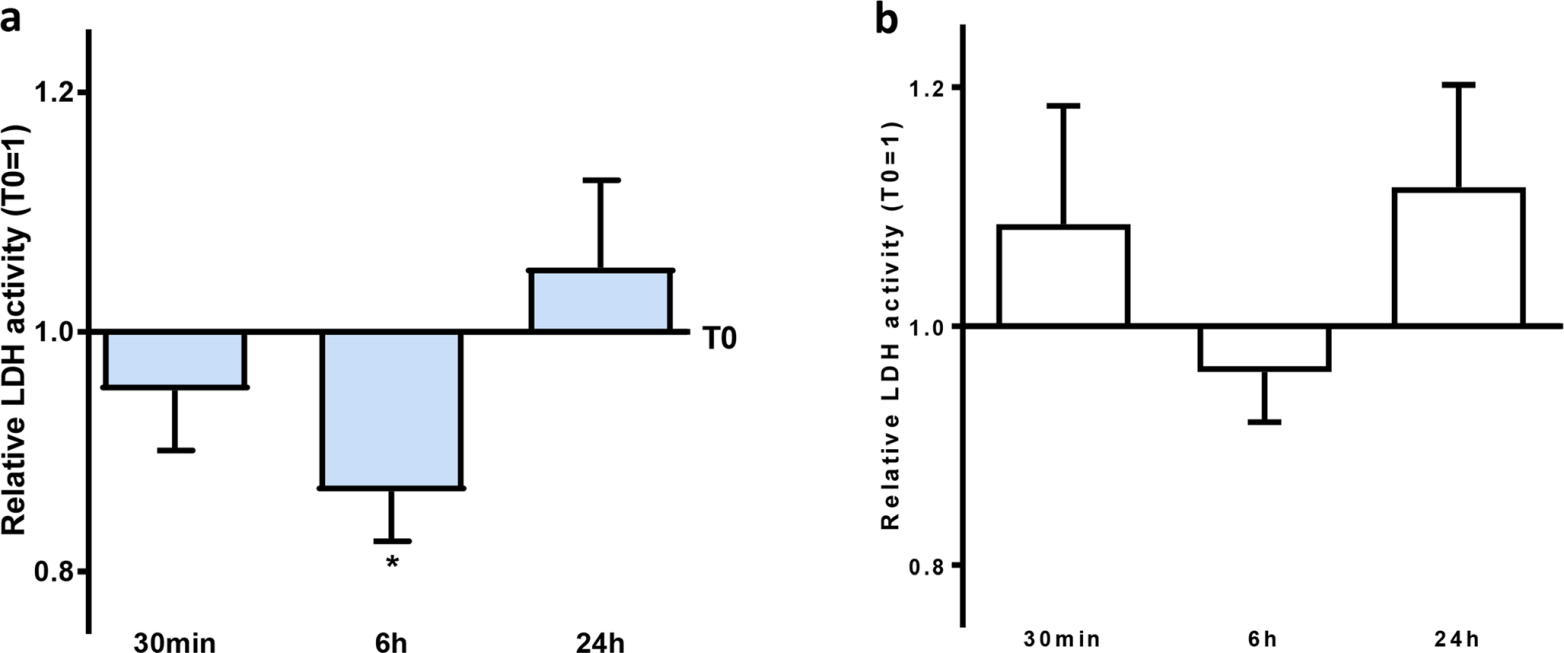
Effect of remote helium-conditioned plasma on hypoxia-induced damage in Huvecs. Quantification of lactate dehydrogenase (LDH) activity as marker of hypoxia-induced cell damage in the supernatant of Huvecs, following 10% plasma incubation for 2 h prior to start of 24-h hypoxia. **a** Relative LDH activity after helium inhalation, baseline (T0) = 1. **b** Relative LDH activity after control gas inhalation, baseline (T0) = 1. Columns represent means (± 95% confidence interval (CI)). *p < 0.05 compared with baseline

The effect of hypoxia-induced cell damage was evaluated by LDH activity, and mean LDH activity of the baseline sample (T0) was used as reference (set as 1.0). We performed experiments using two different plasma concentrations (5% and 10%) with a prehypoxic incubation time of 1 h, but observed no protection against hypoxia (see supplementary data S1). After prolongation of the prehypoxic incubation time to 2 h (using 10% plasma in cell culture medium), we demonstrated cell protection: plasma obtained 6 h after helium treatment (T2) mean 95% CI [lower limit, upper limit] 0.86 [0.77; 0.95] reduced hypoxia-induced cell damage compared with baseline (= reference) plasma, *p* < 0.05. Plasma obtained at the other time points, i.e. directly after helium inhalation (T1) and 24 h after helium inhalation (T3), did not protect HUVEC cells against hypoxia (see Fig. 2a, b). Plasma obtained after treatment with normal air inhalation did not affect hypoxia-induced cell damage in normal and prolonged exposure, for both 5% and 10% concentrations (see online supplement, S2).

### The Role of Caveolin-1 in Remote Helium Protection Against Hypoxia

**Fig. 3:**
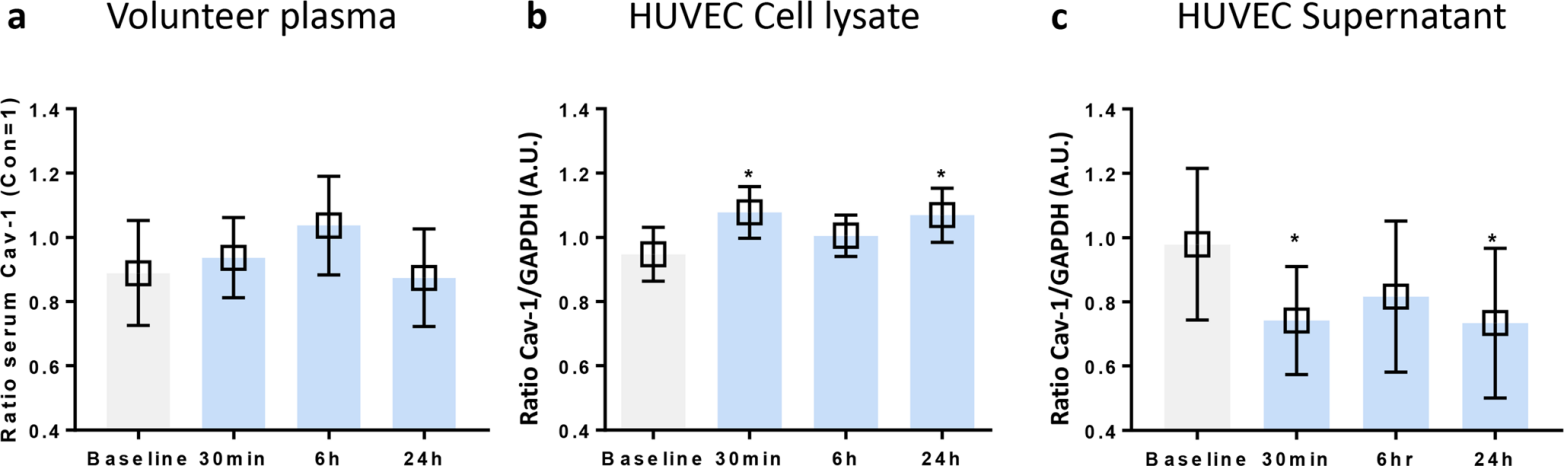
Effect of remote helium conditioning on caveolin-1 levels in HUVEC and plasma from volunteers. **a** ELISA results of caveolin-1 in plasma from volunteers at different time points. **b** Summarised western blot results of caveolin-1 in cell lysate of Huvecs following 24 h of hypoxia. **c** Summarised western blot results of caveolin-1 in supernatant of Huvecs subjected to 24 h of hypoxia. Columns represent means (± 95% CI). *p < 0**.**05 to baseline

In the plasma of the volunteers, the caveolin-1 levels were low, and no significant differences between time points were observed (Fig. 3). Cellular levels of caveolin-1 in HUVECs were measured by western blot analysis and corrected for loading control GAPDH levels. In comparison with baseline, plasma obtained directly after (T1) and 24 h after (T3) helium inhalation caused a significant increase in cellular caveolin levels T0 = 0.95 [0.86, 1.03], T1 = 1.1 [0.99, 1.16] and T3 = 1.0 [0.98, 1.15] respectively, *p* < 0.05. This is shown in Fig. 3b. Surprisingly, plasma obtained 6 h after helium inhalation (T2) did not significantly increase caveolin-1.

In conjunction with increased cellular levels of caveolin, we found decreased levels in the supernatant corresponding time points T1 and T3 compared with baseline T0 = 1.0 [0.74, 1.20], T1 = 0.8 [0.59, 1.02] and T3 = 0.7 [0.50, 0.96], *p* < 0.05, Fig. 3c. Additionally, a lack of significant decrease at T2 matched the results of the cytosol, also shown in Fig. 3b. Hypoxia itself (24 h) did not affect caveolin-1 levels in HUVECs (data not shown).

### Cav-1 Transfection Abolishes Remote Helium-Conditioned Plasma Protection in HUVECs Exposed to Hypoxia

**Fig. 4:**
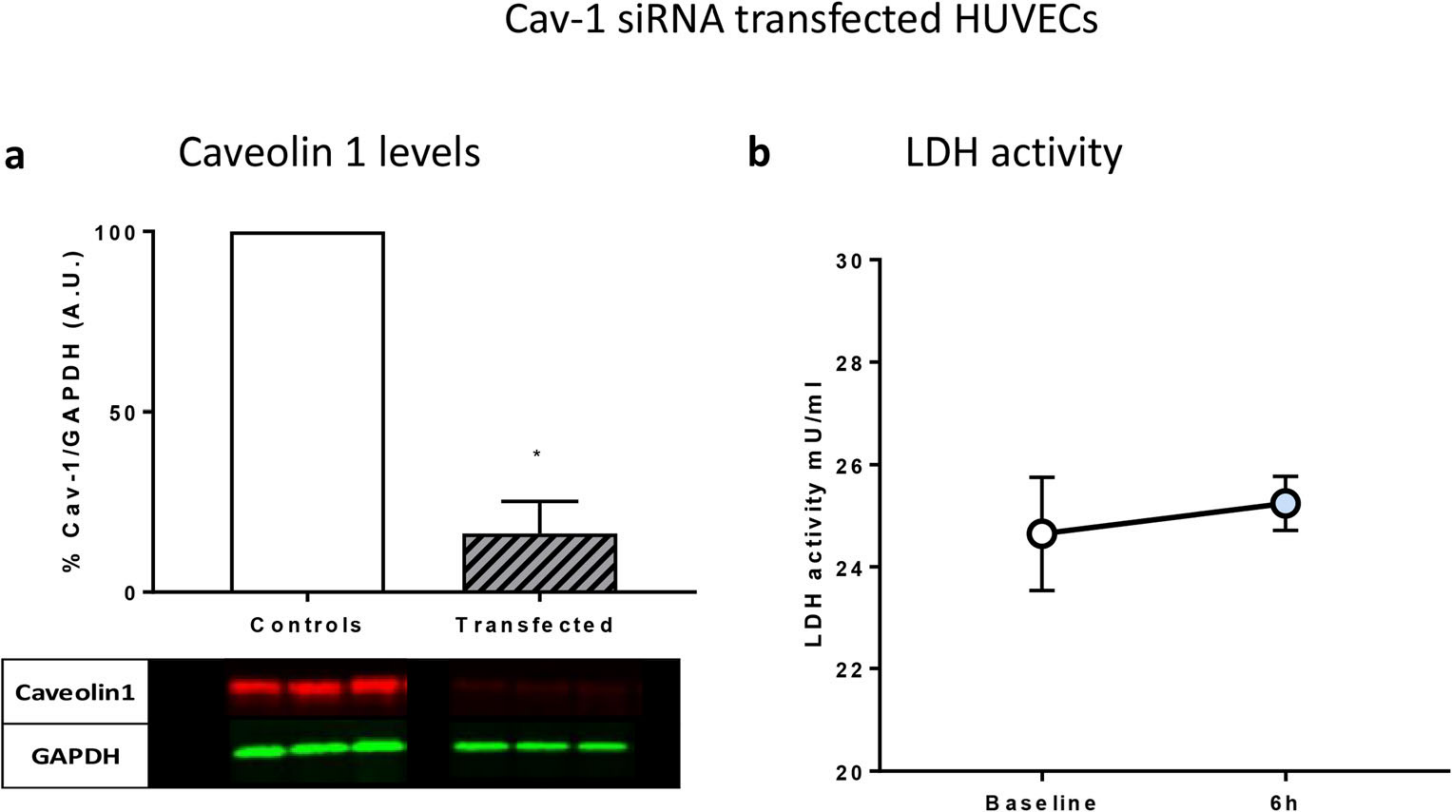
Cav-1 transfection abolishes remote helium-conditioned plasma protection in Huvecs exposed to hypoxia. **a** Levels of cellular Cav-1 in Huvecs transfected with Cav-1 siRNA (16%) and negative control siRNA (100%). Columns represent means (± 95% CI). **b** Absolute LDH activity of Cav-1-transfected Huvecs incubated with plasma from baseline and 6 h after helium treatment. Columns represent means (± 95% confidence interval (CI)). *p < 0.05 compared with baseline. Huvecs human umbilical vein endothelial cells

Transfection with Cav-1 siRNA successfully lowers Cav-1 levels in Huvecs to 16% compared with Huvecs transfected with negative control siRNA (Fig. 4b). In Cav-1 siRNA-transfected Huvecs, the protective effect of T2 plasma was abolished, showing no difference between LDH in cells treated with baseline (T0) plasma (25.5 [24.7, 25.8] and 24.6 [23.5, 25.7]) respectively (Fig. 4a). This indicates that Cav-1 possibly plays a role in helium remote protection against hypoxia.

### The Role of Regulation Kinases in Remote Helium Protection Against Hypoxia

**Fig. 5:**
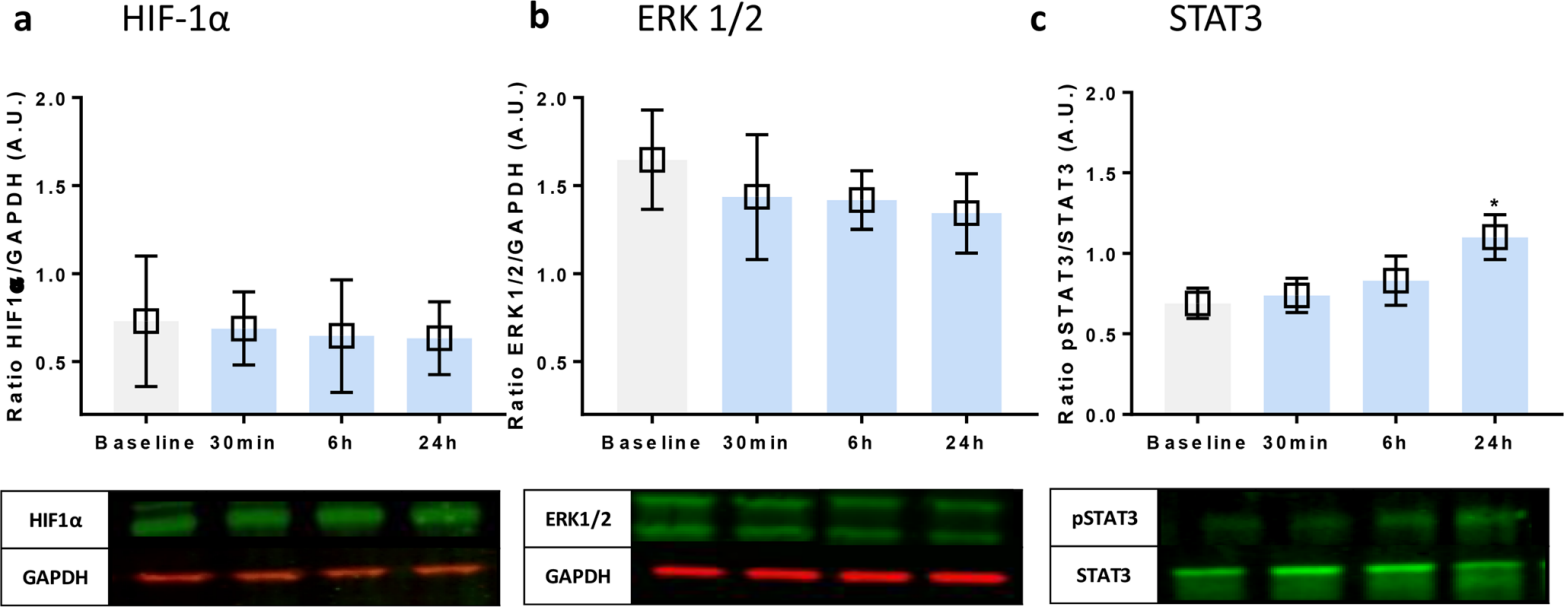
Effect of remote helium conditioning on signal transduction kinases in Huvecs. **a** Western blot results of the ratio of HIF1α compared with GAPDH loading controls in cell lysate of Huvecs following 24 h of hypoxia. **b** Western blot results of the ratio of ERK1/2 and loading control GAPDH in cell lysate of Huvecs following 24 h of hypoxia. **c** Western blot results of activated (phosphorylated to total STAT3) STAT3 in cell lysate of Huvecs following 24 h of hypoxia. Columns represent means (± 95% CI). *p < 0**.**05 to baseline

The levels of HIF1α were determined in the cytosolic fraction. No differences in the HIF1α levels were observed after incubation with plasma from any of the time points when compared with baseline; see Fig. 4a. The levels of ERK1/2 were determined in a similar fashion, and no differences were observed after incubation with helium plasma; see Fig. 5b. The ratio of activated (phosphorylated to total (p/t)) STAT3 showed a significant increase after incubation with plasma obtained 24 h after helium inhalation compared with baseline T0 = 0.7 [0.49, 0.89], T3 = 1.1 [0.81, 1.39], *p* < 0.05. None of the other time points showed increased levels of activated STAT3, Fig. 5c.

### The Role of eNOS in Remote Helium Protection Against Hypoxia

**Fig. 6:**
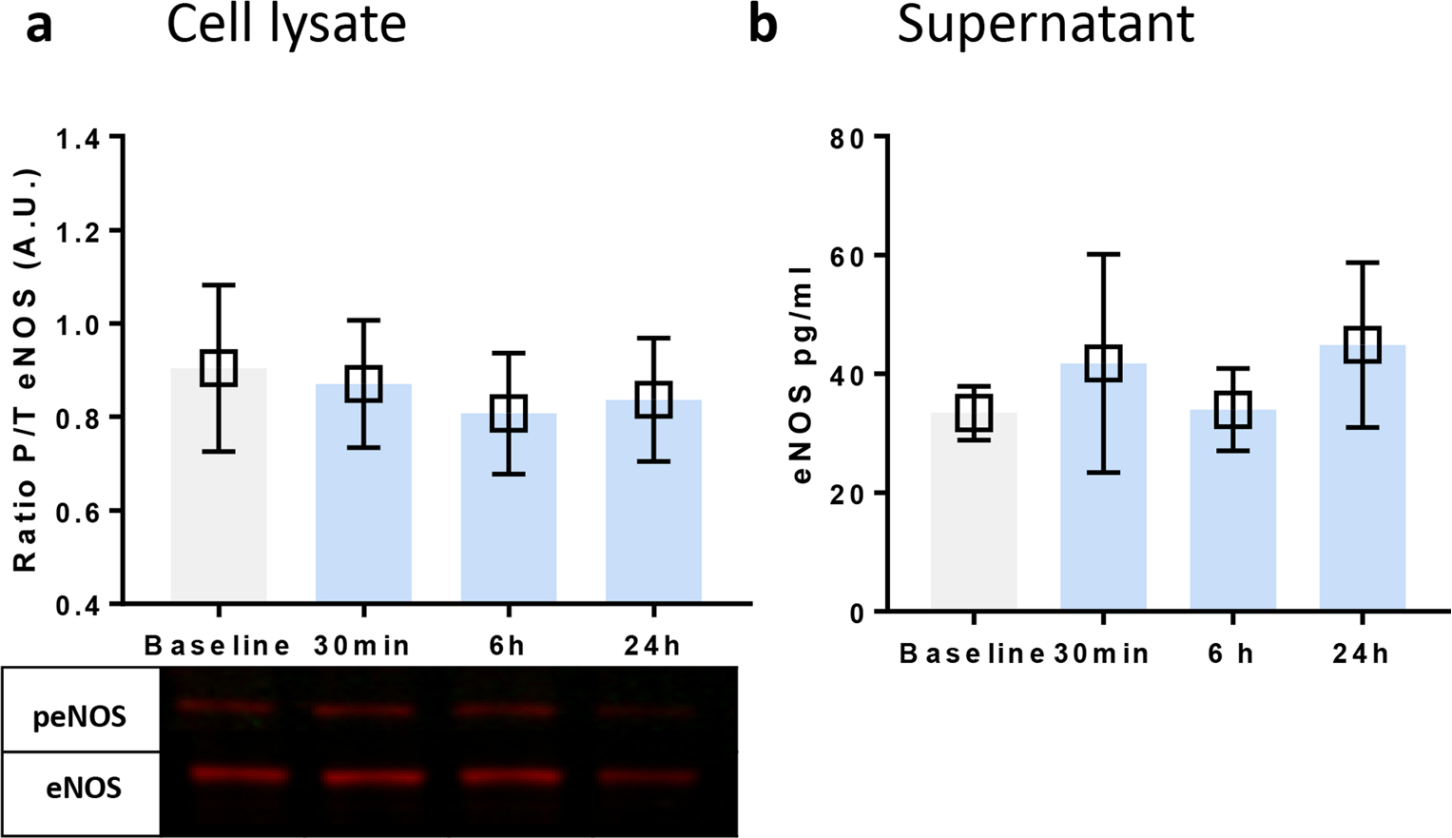
The role of eNOS in remote helium conditioning. **a** Western blot results of the ratio of activated (phosphorylated to total) eNOS in Huvecs after prehypoxic exposure to plasma. **b** Results of ELISA measurements of eNOS in the supernatant of Huvecs after prehypoxic exposure to plasma. Columns represent means (± 95% CI). *p < 0**.**05 to baseline

In the plasma of the volunteers, levels of eNOS were below the detection limit. In the supernatant of the Huvecs exposed to 24 h of hypoxia, eNOS levels were also undetectable. After incubation with plasma, but before start of hypoxia, cells and supernatant were collected and analysed using western blot (cells) and ELISA (supernatant). No differences were observed in eNOS levels; see Fig. 6a, b.

## Discussion

This is the first study showing that helium induces remote protection by using plasma from healthy helium-breathing volunteers. The respective plasma decreased hypoxia-induced cell damage in Huvecs in vitro. Thus, in addition to in vivo protection in humans [2], we now show that helium induces remote conditioning by protecting endothelial cells in vitro against hypoxia.

When compared with RIPC plasma, helium plasma did not induce protection against hypoxia-induced cell damage in a concentration of 5% [18]. Increasing the plasma concentration to 10% did not result in protection either, yet a prolonged prehypoxic exposure (2 h) of 10% helium plasma protected endothelial cells against hypoxia. This suggests that helium plasma is less potent to induce protection against hypoxia than RIPC plasma. A possible explanation could be that helium was applied as a continuous stimulus, and preconditioning is generally more pronounced after repetitive stimuli [21]. Further research is needed to elucidate whether the stimulus by helium inhalation can be optimised and the protective potency of helium plasma can be increased.

Surprisingly, helium plasma obtained 6 h after helium inhalation did not show increased caveolin-1 levels in the cells after hypoxia. This in contrast to the fact that helium plasma from this time point protected the cells against hypoxia-induced cell damage (as measured by LDH activity levels). Unfortunately, no other data are available regarding caveolin levels in endothelium following remote conditioning.

This lack of caveolin-1 increase after helium-containing plasma obtained after 6 h can be considered in two different ways: first, one can conclude that helium conditioning is not mediated by caveolin-1, and consider the increased levels of caveolin-1 after exposure to plasma obtained after 30 min and 24 h as epiphenomena and unrelated to helium conditioning. However, considering the fact that caveolin is strictly balanced and continuously regulated, we hypothesised that increased caveolin-1 levels have preceded the actual protection we demonstrated in the plasma taken 6 h after helium inhalation. To test this, we repeated the experiment in caveolin-1 siRNA-transfected Huvecs and showed that the protective effect of T2 plasma was abolished. This could indicate that caveolin-1 plays a role in helium remote protection against hypoxia.

### Caveolins and Remote Preconditioning

Three different caveolin isomeres are present, all with different origin and functions. Caveolin-3 typically originates from cardiomyocytes, whereas caveolin-1 is mostly found in endothelial cells. A previous study showed that helium treatment of endothelial cells lowers levels of caveolin-1 in Huvecs and increases levels of circulating caveolin-1 in the supernatant of these cells [19]. Additionally, helium postconditioning leads to increased levels of circulating caveolin-3, as well as increased levels of caveolin-1 and -3 in the myocardial area at risk [22]. Considering these data, we expected that helium would result in increased circulating levels of caveolin-1 in blood. However, the data from the current study do not support this hypothesis. This could either mean that circulating levels of caveolin-1 are not the mediating factor in helium remote preconditioning, or the circulating amounts were below the detection limit. Our measurement could have been influenced by dilution of caveolin-1 in the systemic circulation.

Furthermore, caveolins are primarily concentrated in the cholesterol-enriched membranes, with reports indicating that cell stress can induce the translocation of caveolins from plasma membranes to mitochondrial membranes and other non-caveolae membranes [23]. Measurements of caveolin in membrane/particulate fractions could have yielded different results, but were not measured during the experiments. Therefore, definitive conclusions should only be drawn very carefully.

Caveolins are not only involved in conveying a remote conditioning signal, but also in mediating an effect within the target organ. Ischemic and anaesthetic preconditioning in cardiomyocytes involved increased levels of caveolin-3 in the target organ [16, 24]. Protection of ischemic preconditioning was abrogated when caveolin-3 was absent in caveolin-3 knockout mice, proving that caveolin in the target organ is essential [16, 25]. In the present study, we showed increased levels of caveolin-1 in Huvecs after exposure to helium plasma obtained 30 min and 24 h after helium treatment. Under normal circumstances, caveolin regulates endocytosis and exocytosis in cells, is continuously recycled, and total levels of caveolins are balanced [26, 27]. We found that helium plasma increased the caveolin-1 cellular level and decreased the buoyant level, possibly indicating that less caveolin-1 is secreted to the supernatant by exocytosis. Additional experiments investigating the effect of 2 h of prehypoxic exposure to 10% helium plasma without hypoxia showed lowered caveolin-1 levels, but not significantly (supplemental data S3). This indicates that hypoxia, or the damaging factor, is needed to reveal upregulation of caveolin-1. Hypoxia itself does not increase caveolin-1 levels because caveolin-1 levels after exposure to baseline plasma remained unaffected.

### Remote Helium Preconditioning and the RISK and SAFE Pathway

The role of STAT3 and STAT5 activation in RIPC is not entirely clear and appears to be dependent of species, targeted organs and cell types in vitro [28, 29]. In the present study, we demonstrated that plasma obtained 24 h after helium inhalation activated STAT3 in comparison with baseline. None of the other time points activated STAT3. These data suggest that helium remote protection is not mediated by STAT3 activation.

Prolonged hypoxia (24 h) previously caused activation of STAT3 by regulating HIF1α in Huvecs [30]. The difference between these studies is the presence of human plasma, which could have influenced STAT3 activation. Our data show that helium plasma obtained 24 h after inhalation activates STAT3 and could be a possible mediator of the late phase of helium preconditioning which was demonstrated before [2].

RIPC plasma induces protection against hypoxia, in which upregulation of HIF1α and activation of ERK1/2 is involved [18]. In contrast to these findings, the protection against hypoxia from helium plasma does not involve upregulation of HIF1α and ERK1/2. Yet another example of different mechanisms of action is ERK1/2, Akt [31], GSK3B and PKC-ε [32], all of which are involved in (remote) ischemic preconditioning, but not in helium remote conditioning.

These data indicate that helium remote conditioning involves a different set of mediators that are not linked to the RISK pathway but rather to caveolin secretion and extracellular vesicle formation.

Very recent evidence from the literature links extracellular vesicle release to the protection of the heart [33]. Regarding helium and organ protection, a recent in vitro study of our group showed that helium treatment induces the release of caveolin into the supernatant of endothelial cells [19]. Interestingly, the supernatant of these cells, which contained increased levels of caveolin, was able to reduce the permeability of another set of remote endothelial cells. These findings, together with another earlier study showing that helium exposure could increase microparticle production in endothelial cells [34], might support the hypothesis that the production of microparticles might be linked or even mediated via caveolin levels in the supernatant and might be in part responsible for the observed effects of helium [19].

Nanoparticle tracking analysis of the helium-treated supernatant indeed showed an increased amount of particles released after 6 h compared with control gas [19]. This effect was present after 6 h, was reduced after 12 h and even abolished after 24 h. Because of the timing and the size of the particles (caveolin can produce varying vesicles from 50 to 100 nm in size [35]), the data suggest that these particles are caveolin-1 related, although this cannot be proven with our current experiments.

Thus even though the timing of the increase matches our present results, further research is needed to confirm this possible connection in humans in vivo.

### Caveolin-1 and eNOS

Regulation of eNOS is complex and negatively regulated by caveolin-1 [36]. After 24 h of hypoxia, levels of eNOS in the supernatant were below detection limit. This concurs with previous research demonstrating that hypoxia decreases eNOS activation in HUVECs but not in arterial endothelial cells [37]. Exposure to helium plasma for 2 h without hypoxia showed no significant increases in eNOS levels. In this study, quiescent HUVECs were used in a model without flow, and we cannot exclude that this influenced our results [38, 39].

Weakness of this study is the fact that we used plasma from healthy volunteers, and translating preconditioning from healthy volunteers to a clinical setting has been proven difficult. Besides this, in vitro protection of endothelial cells does not mean that in vivo protection of endothelial cells is also possible.

It has to be considered that at the time of the protection against hypoxia (6 h), no effect in the cells on caveolin levels is seen, only before and after this time point. Our previous research showed a matching timeframe for increased caveolin in the supernatant [19], but did not focus on protection against hypoxia. These previous findings led to our hypothesis that increased caveolin-1 levels have preceded the actual protection, and the additional knockdown experiments confirmed this.

In conclusion, helium has the capacity to induce remote conditioning in humans and the ability to protect remote organs against ischemia-reperfusion injury. Helium is easy to administer, readily available and has no side effects. Helium, alone or in combination with other conditioning strategies, could be a promising conditioning agent to reduce ischemia-reperfusion injury in a clinical setting.

## Electronic Supplementary Material

ESM 1 (DOC 66 kb)

## References

[1] Pagel PS, Krolikowski JG, Shim YH, Venkatapuram S, Kersten JR, Weihrauch D, Warltier DC, Pratt PF (2007). Noble gases without anesthetic properties protect myocardium against infarction by activating prosurvival signaling kinases and inhibiting mitochondrial permeability transition in vivo. Anesth Analg.

[2] Smit KF, Oei GT, Brevoord D, Stroes ES, Nieuwland R, Schlack WS, Hollmann MW, Weber NC, Preckel B (2013). Helium induces preconditioning in human endothelium in vivo. Anesthesiology..

[3] Preckel B, Mullenheim J, Moloschavij A, Thamer V, Schlack W (2000). Xenon administration during early reperfusion reduces infarct size after regional ischemia in the rabbit heart in vivo. Anesth Analg.

[4] Weber NC, Stursberg J, Wirthle NM, Toma O, Schlack W, Preckel B (2006). Xenon preconditioning differently regulates p44/42 MAPK (ERK 1/2) and p46/54 MAPK (JNK 1/2 and 3) in vivo. Br J Anaesth.

[5] Weber NC, Toma O, Wolter JI, Obal D, Mullenheim J, Preckel B, Schlack W (2005). The noble gas xenon induces pharmacological preconditioning in the rat heart in vivo via induction of PKC-epsilon and p38 MAPK Br. Aust J Pharm.

[6] Pagel PS, Krolikowski JG, Pratt PF, Shim YH, Amour J, Warltier DC, Weihrauch D (2008). Inhibition of glycogen synthase kinase or the apoptotic protein p53 lowers the threshold of helium cardioprotection in vivo: the role of mitochondrial permeability transition. Anesth Analg.

[7] Pagel PS, Krolikowski JG, Amour J, Warltier DC, Weihrauch D (2009). Morphine reduces the threshold of helium preconditioning against myocardial infarction: the role of opioid receptors in rabbits. J Cardiothorac Vasc Anesth.

[8] Pagel PS, Krolikowski JG, Pratt PF, Shim YH, Amour J, Warltier DC, Weihrauch D (2008). Reactive oxygen species and mitochondrial adenosine triphosphate-regulated potassium channels mediate helium-induced preconditioning against myocardial infarction in vivo. J Cardiothorac Vasc Anesth.

[9] Heinen A, Huhn R, Smeele KM, Zuurbier CJ, Schlack W, Preckel B, Weber NC, Hollmann MW (2008). Helium-induced preconditioning in young and old rat heart: impact of mitochondrial Ca(2+)-sensitive potassium channel activation. Anesthesiology.

[10] Huhn R, Weber NC, Preckel B, Schlack W, Bauer I, Hollmann MW, Heinen A (2012). Age-related loss of cardiac preconditioning: impact of protein kinase A. Exp Gerontol.

[11] Pike LJ, Han X, Chung KN, Gross RW (2002). Lipid rafts are enriched in arachidonic acid and plasmenylethanolamine and their composition is independent of caveolin-1 expression: a quantitative electrospray ionization/mass spectrometric analysis. Biochemistry.

[12] Fridolfsson HN, Roth DM, Insel PA, Patel HH (2014). Regulation of intracellular signaling and function by caveolin. FASEB J.

[13] Balligand JL, Feron O, Dessy C (2009). eNOS activation by physical forces: from short-term regulation of contraction to chronic remodeling of cardiovascular tissues. Physiol Rev.

[14] Ballard-Croft C, Locklar AC, Kristo G, Lasley RD (2006). Regional myocardial ischemia-induced activation of MAPKs is associated with subcellular redistribution of caveolin and cholesterol. Am J Physiol Heart Circ Physiol.

[15] Krajewska WM, Maslowska I (2004). Caveolins: structure and function in signal transduction cell. Mol Biol Lett.

[16] Patel HH, Tsutsumi YM, Head BP, Niesman IR, Jennings M, Horikawa Y, Huang D, Moreno AL, Patel PM, Insel PA, Roth DM (2007). Mechanisms of cardiac protection from ischemia/reperfusion injury: a role for caveolae and caveolin-1. FASEB J.

[17] Przyklenk K, Bauer B, Ovize M, Kloner RA, Whittaker P (1993). Regional ischemic ‘preconditioning’ protects remote virgin myocardium from subsequent sustained coronary occlusion. Circulation.

[18] Weber NC, Riedemann I, Smit KF, Zitta K, van de Vondervoort D, Zuurbier CJ, Hollmann MW, Preckel B, Albrecht M (2015). Plasma from human volunteers subjected to remote ischemic preconditioning protects human endothelial cells from hypoxia-induced cell damage. Basic Res Cardiol.

[19] Smit KF, Konkel M, Kerindongo R, Landau MA, Zuurbier CJ, Hollmann MW, Preckel B, Nieuwland R, Albrecht M, Weber NC (2018). Helium alters the cytoskeleton and decreases permeability in endothelial cells cultured in vitro through a pathway involving Caveolin-1. Sci Rep.

[20] Smit KF, Brevoord D, De Hert S, de Mol BA, Kerindongo RP, van Dieren S, Schlack WS, Hollmann MW, Weber NC, Preckel B (2016). Effect of helium pre- or postconditioning on signal transduction kinases in patients undergoing coronary artery bypass graft surgery. J Transl Med.

[21] Bein B, Renner J, Caliebe D, Hanss R, Bauer M, Fraund S, Scholz J (2008). The effects of interrupted or continuous administration of sevoflurane on preconditioning before cardio-pulmonary bypass in coronary artery surgery: comparison with continuous propofol. Anaesthesia.

[22] Flick M, Albrecht M, Oei GT, Steenstra R, Kerindongo RP, Zuurbier CJ, Patel HH, Hollmann MW, Preckel B, Weber NC (2016). Helium postconditioning regulates expression of caveolin-1 and -3 and induces RISK pathway activation after ischaemia/reperfusion in cardiac tissue of rats. Eur J Pharmacol.

[23] Wang J, Schilling JM, Niesman IR, Headrick JP, Finley JC, Kwan E, Patel PM, Head BP, Roth DM, Yue Y, Patel HH (2014). Cardioprotective trafficking of caveolin to mitochondria is Gi-protein dependent. Anesthesiology.

[24] Hamaguchi E, Tanaka K, Tsutsumi R, Sakai Y, Fukuta K, Kasai A, Tsutsumi YM (2015). Exendin-4, glucagon-like peptide-1 receptor agonist, enhances isoflurane-induced preconditioning against myocardial infarction via caveolin-3 expression. Eur Rev Med Pharmacol Sci.

[25] Horikawa YT, Patel HH, Tsutsumi YM, Jennings MM, Kidd MW, Hagiwara Y, Ishikawa Y, Insel PA, Roth DM (2008). Caveolin-3 expression and caveolae are required for isoflurane-induced cardiac protection from hypoxia and ischemia/reperfusion injury. J Mol Cell Cardiol.

[26] Parton RG, del Pozo MA (2013). Caveolae as plasma membrane sensors, protectors and organizers. Nat Rev Mol Cell Biol.

[27] Pelkmans L, Burli T, Zerial M, Helenius A (2004). Caveolin-stabilized membrane domains as multifunctional transport and sorting devices in endocytic membrane traffic. Cell.

[28] Heusch G, Musiolik J, Kottenberg E, Peters J, Jakob H, Thielmann M (2012). STAT5 activation and cardioprotection by remote ischemic preconditioning in humans: short communication. Circ Res.

[29] Janmaat ML, Heerkens JL, de Bruin AM, Klous A, de Waard V, de Vries CJ (2010). Erythropoietin accelerates smooth muscle cell-rich vascular lesion formation in mice through endothelial cell activation involving enhanced PDGF-BB release. Blood.

[30] Dal Monte M, Martini D, Ristori C, Azara D, Armani C, Balbarini A, Bagnoli P (2011). Hypoxia effects on proangiogenic factors in human umbilical vein endothelial cells: functional role of the peptide somatostatin. Naunyn Schmiedeberg's Arch Pharmacol.

[31] Huhn R, Heinen A, Weber NC, Kerindongo RP, Oei GT, Hollmann MW, Schlack W, Preckel B (2009). Helium-induced early preconditioning and postconditioning are abolished in obese Zucker rats in vivo. J Pharmacol Exp Ther.

[32] Oei GT, Huhn R, Heinen A, Hollmann MW, Schlack WS, Preckel B, Weber NC (2012). Helium-induced cardioprotection of healthy and hypertensive rat myocardium in vivo. Eur J Pharmacol.

[33] Sluijter JPG, Davidson SM, Boulanger CM, Buzas EI, de Kleijn DPV, Engel FB, Giricz Z, Hausenloy DJ, Kishore R, Lecour S, Leor J, Madonna R, Perrino C, Prunier F, Sahoo S, Schiffelers RM, Schulz R, Van Laake LW, Ytrehus K, Ferdinandy P (2018). Extracellular vesicles in diagnostics and therapy of the ischaemic heart: Position Paper from the Working Group on Cellular Biology of the Heart of the European Society of Cardiology. Cardiovasc Res.

[34] Smit KF, Kerindongo RP, Boing A, Nieuwland R, Hollmann MW, Preckel B, Weber NC (2015). Effects of helium on inflammatory and oxidative stress-induced endothelial cell damage Exp. Cell Res.

[35] Li S, Galbiati F, Volonte D, Sargiacomo M, Engelman JA, Das K, Scherer PE, Lisanti MP (1998). Mutational analysis of caveolin-induced vesicle formation. Expression of caveolin-1 recruits caveolin-2 to caveolae membranes. FEBS Lett.

[36] Dudzinski DM, Michel T (2007). Life history of eNOS: partners and pathways. Cardiovasc Res.

[37] Krause BJ, Prieto CP, Munoz-Urrutia E, San Martin S, Sobrevia L, Casanello P (2012). Role of arginase-2 and eNOS in the differential vascular reactivity and hypoxia-induced endothelial response in umbilical arteries and veins. Placenta.

[38] Sud N, Kumar S, Wedgwood S, Black SM (2009). Modulation of PKCdelta signaling alters the shear stress-mediated increases in endothelial nitric oxide synthase transcription: role of STAT3. Am J Phys Lung Cell Mol Phys.

[39] Sathanoori R, Bryl-Gorecka P, Muller CE, Erb L, Weisman GA, Olde B, Erlinge D (2017). P2Y2 receptor modulates shear stress-induced cell alignment and actin stress fibers in human umbilical vein endothelial cells. Cell Mol Life Sci.

