# Supplementary material for: Plasma from Volunteers Breathing Helium Reduces Hypoxia-Induced Cell Damage in Human Endothelial Cells—Mechanisms of Remote Protection Against Hypoxia by Helium
Source: Cardiovasc Drugs Ther. 2019 Apr 25;33(3):297–306. doi: 10.1007/s10557-019-06880-2 (PMC6538579; doi:10.1007/s10557-019-06880-2)
Supplement: Supplementary file 1 — (DOC 66 kb) [file 10557_2019_6880_MOESM1_ESM.doc]

**Supplemental data**

**FigS1**

**
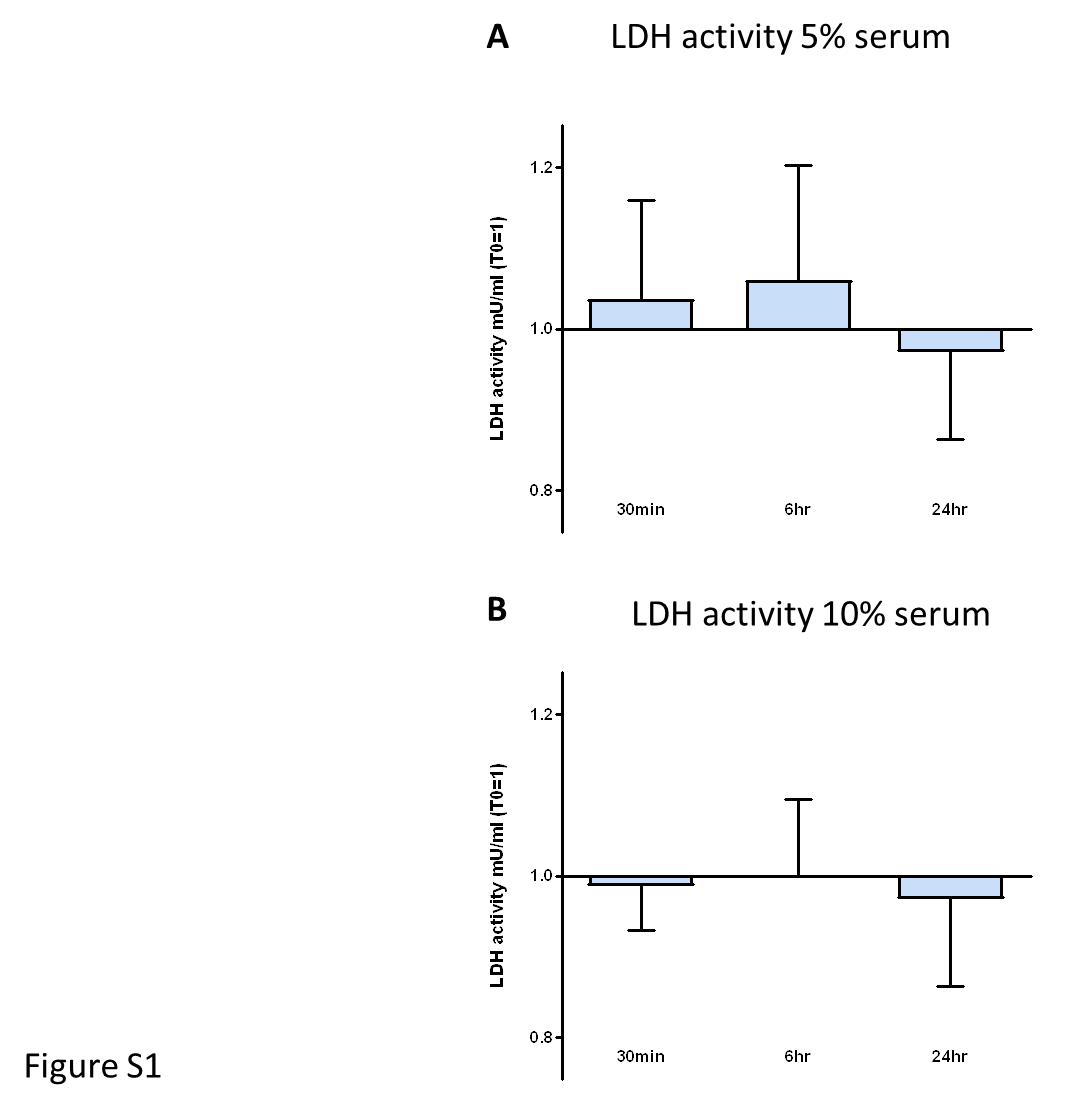
**

**Supplemental data S1**

**Exposure to 5% helium plasma and 10% helium plasma for 1 hour only, does not protect against hypoxia induced damage in HUVEC.**

Quantification of LDH activity in the supernatant of HUVEC after 24 hours of hypoxia as a marker of hypoxia induced cell damage. Cells were incubated with plasma collected at baseline (T0) = 1.0, 30 minutes (T1), 6 hours (T2) and 24 hours (T3) after helium treatment.

Panel A shows results of LDH activity after incubation with 5% helium plasma and 24 hours of hypoxia. No differences were observed between timepoints. Panel B shows results following incubation with 10% helium plasma and 24 hours of hypoxia. No differences were observed between timepoints.

**FigS2**

**
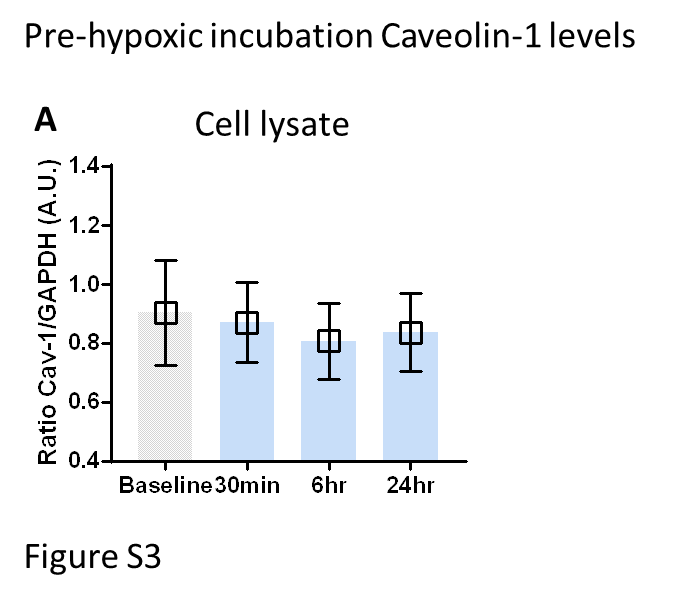
**

**Supplemental data S2**

**Caveolin-1 levels after pre hypoxic incubation of helium plasma**

This graph shows the levels of caveolin-1 in cytosol of HUVEC after exposure to 10% helium plasma without hypoxia.

Columns represent means and 95% CI.
